# Identification of two anti-*Candida* antibodies associated with the survival of patients with candidemia

**DOI:** 10.1128/mbio.02769-23

**Published:** 2023-12-13

**Authors:** Carla Bromuro, Brunella Posteraro, Rita Murri, Massimo Fantoni, Mario Tumbarello, Maurizio Sanguinetti, Rosanna Dattilo, Roberto Cauda, Antonio Cassone, Antonella Torosantucci

**Affiliations:** 1 Dipartimento di Malattie Infettive, Istituto Superiore di Sanità, Rome, Italy; 2 Dipartimento di Scienze Biotecnologiche di Base, Cliniche Intensivologiche e Perioperatorie, Università Cattolica del Sacro Cuore, Policlinico Universitario A. Gemelli IRCCS, Rome, Italy; 3 Dipartimento Salute e Bioetica, Sezione Malattie Infettive, Università Cattolica del Sacro Cuore, Policlinico Universitario A. Gemelli IRCCS , Rome, Italy; 4 Dipartimento Biotecnologie Mediche, University of Siena, Siena, Italy; 5 Polo d'Innovazione della Genomica, Genetica e Biologia, Siena, Italy; Texas Christian University, Fort Worth, Texas, USA

**Keywords:** candidemia, antibody immunity, protection, patient survival

## Abstract

We retrospectively studied antibody immunity in 92 candidemia patients, using sera taken at candidemia diagnosis. All patients showed the presence of IgG antibodies against all tested *Candida* antigens, namely Als3, Mp65, Hyr1 and Eno1, at levels significantly higher than those of non-candidemia controls. Both correlation and multivariable logistic regression analyses showed that the antibodies against Als3 and-Mp65, two known immunodominant and virulence-related proteins, were significantly associated with lower 30-day patient mortality. In particular, high titers of anti-Als3 antibodies were associated with survival in all subgroups of frail, more critical patients (over-median aged, infected by *C. albicans* or with septic shock) while high anti-Mp65 IgG levels were associated with survival in younger patients and in those infected by non-*albicans Candida* species or showing no signs of septic shock. A multivariable logistic model including anti-Als3 or anti-MP65 antibody levels and other variables, such as the serum β-glucan level at candidemia diagnosis, septic shock occurrence, the presence of diabetes, and initial antifungal treatment with fluconazole, was highly predictive of candidemia outcome, with an area under the curve of 0.85–0.86. Overall, the data demonstrate the ability of candidemia patients to mount a sustained memory antibody response to *Candida* antigens and that some of these responses have a sizeable impact on patient survival. Our data invite consideration of a multicenter, prospective investigation of antibody immunity in candidemia.

**IMPORTANCE:**

Candidemia (bloodstream invasion by *Candida* species) is a major fungal disease in humans. Despite the recent progress in diagnosis and treatment, therapeutic options are limited and under threat of antimicrobial resistance. The disease mortality remains high (around 40%). In contrast with deep-seated invasive candidiasis, particularly that occurring in patients with hematologic malignancies and organ transplants, patients with candidemia are often not immunocompromised and therefore able to mount memory anticandidal immune responses, perhaps primed by *Candida* commensalism. We investigated antibody immunity in candidemia patients and report here on the ability of these patients to produce antibodies that react with *Candida* antigens. In particular, the patients with high titers of IgG reactive with two immunodominant, virulence-associated antigens (Als3 and MP65) had a higher 30-day survival. If confirmed by controlled, prospective clinical studies, our data could inform the development of antibody therapy to better treat a severe fungal infection such as candidiasis.

## INTRODUCTION

Invasive diseases caused by *Candida albicans* and other *Candida* species remain highly prevalent among hospitalized patients, despite progress in diagnosis and therapy. Aged subjects with comorbidities, organ transplants, immunosuppression or intensive care are especially afflicted by elevated mortality upon infection by the above fungi (1).

In the setting of invasive candidiasis, candidemia (bloodstream invasion) is of particular interest for its relatively high incidence and mortality in patients undergoing deep organ surgery and bearing central venous catheters but non-neutropenic and without other manifest immunodeficiencies (2, 3). As such, most subjects with candidemia could in principle be helped to control the invasive fungal agent by some forms of immune interventions, taking into consideration that the available effective therapeutics are few and continue to suffer resistance phenomena (1, 4). Of interest, there are several reports of efficacious antibody treatments in experimental models of both local and systemic *Candida* infections (5). However, too little is known about the immune responses of candidemia patients to envision any kind of realistic intervention.

In a preliminary report, we showed that candidemia patients can raise a marked secondary antibody response to *Candida* polysaccharides and glycoproteins. Antibodies against the protein constituent of a 65 Kdal mannoprotein (Mp65), an immunodominant recall antigen of *Candida albicans* (6, 7), were associated with lower 30-day mortality (8).

In this paper, we have extended our initial observation to the evaluation of antibody immunity to other fungal antigens chosen among those that previous experimental studies in animal models suggested being potentially involved in the protection from *Candida* infections. Correlation and multivariable logistic regression analyses were used to assess the relevance and potential impact of antibodies on candidemia outcome along with demographic, clinical and treatment variables. Here, we report that antibodies against another well-studied fungal antigen, the Als3 adhesin (9), are present at high titers in subjects with candidemia and, as those against MP65, are independently associated with lower mortality in our patient cohort.

## RESULTS

### Candidemia patients

Ninety-two patients with candidemia, hospitalized from January 2012 to January 2019 at the A. Gemelli Hospital of Rome, Italy (an academic tertiary care center with 1,400 beds and ~50,000 hospital admissions per year), were enrolled (Table 1; see also reference 8). All patients (57 males and 35 females; mean age 68.7 + 15.9; median 72) had clinical symptoms of infection, *Candida*-positive blood cultures, and positive β-glucanemia (>80 pg/mL).

**TABLE 1:** Candidemia patients: demographic, microbiological, and clinical characteristics

	Total	Survivors	Non-survivors^a^	
All patients, *n* (%)	92	54 (58.7)	38 (41.3)	
Male, *n*/total (%)	57/92 (62.0)	32/57 (56.1)	25/57 (43.9)	
Female, *n*/total (%)	35/92 (38.0)	22/35 (62.8)	13/35 (37.2)	
Age, median years (range)	72.0 (14–91)	68.0 (20–88)	76.5 (14–91)	
Median level (range) of serum β-glucan at onset of candidemia^b^, pg/mL	>500 (80–>500)	>500 (80–>500)	>500 (135–>500)	
APACHE II score at first positive blood culture, median (range)	15 (2-37)	12 (2-35)	16.5 (15-37)	
Septic shock, *n*/total (%)	39/92 (42.4)	14/39 (35.9)	25/39 (64.1)	
Central venous catheter, *n*/total (%)	49/92 (53.3)	29/49 (59.2)	20/49 (40.8)	
Surgery, *n*/total (%)	42/92 (45.6)	23/42 (54.8)	19/42 (45.2)	
Hematologic malignancy, n/total (%)	6/92 (6.5)	3/6 (50.0)	3/6 (50.0)	
HIV infection, *n*/total (%)	1/92 (1.1)	0/1 (0)	1/1 (100)	
Treatment with immunosuppressants, *n*/total (%)	10/92 (10.9)	6/10 (60.0)	4/10 (40.0)	
Treatment with steroids, *n*/total (%)	16/92 (17.4)	11/16 (68.7)	5/16 (31.3)	
Treatment with steroids and immunosuppressants, *n*/total (%)	3/92 (3.2)	3/3 (100)	0/3 (0)	
Solid tumor, *n*/total (%)	21/92 (21.4)	12/21 (57.1)	9/21 (42.9)	
Chronic obstructive pulmonary disease, *n*/total (%)	22/92 (23.9)	12/22(54.5)	10/22 (45.5)	
Chronic renal failure, *n*/total (%)	24/92 (26.1)	13/24 (54.2)	11/24 (45.8)	
Diabetes mellitus, *n*/total (%)	17/92 (18.5)	5/17 (29.4)	12/17 (70.6)	
Antibiotic in the 30 days prior to first positive blood culture, *n*/total (%)	74/92 (80.4)	45/74 (60.8)	29/74 (39.2)	
			
Species isolated from the first *Candida*-positive blood culture				
*C. albicans^c^*	47/92 (51.1)	24/47 (51.1)	23/47 (48.9)	
All non-*albicans* species	45/92 (48.9)	30/45 (66.7)	15/45 (33.3)	
*Candida parapsilosis*	22/92 (23.9)	17/22 (77.3)	6/22 (27.3)	
*Candida tropicalis*	9/92 (9.8)	6/9 (66.7)	3/9 (33.3)	
*Candida glabrata*	7/92 (7.6)	4/7 (57.1)	3/7 (42.9)	
			
Other minor species*^d^*	7/92 (7.6)	5/7 (71.4)	2/7 (28.6)	
Initial antifungal therapy^e^				
Anidulafungin	42/92 (45.6)	22/42 (52.4)	20/42 (47.6)	
Caspofungin	30/92 (32.6)	21/30 (70.0)	9/30 (30.0)	
Fluconazole	17/92 (18.5)	8/17 (47.1)	9/17 (52.9)	
Liposomal amphotericin B	3/92 (3.3)	3/3 (100)	0/3 (0)	

^a^ Mortality within 30 days from the first documented episode of candidemia was considered.

^b^ Beta-glucan levels measured in sera from the same blood samples producing the first *Candida*-positive blood culture.

^c^ In four patients, *C. albicans* was isolated together with other species (*C. krusei*, 1; *C. glabrata*, 3).

^d^ *C. guilliermondii*, 3; *C. krusei*, 1; *C. orthopsilosis*, 1; *C. lusitaniae*, 1; *C. robusta*, 1.

^e^ Eighteen patients were treated with more than one antifungal during the infection.

Their median Acute Physiology and Chronic Health Evaluation II (APACHE II) score at the time of the first *Candida*-positive blood culture was 15 (range: 2–37). About half of the patients had undergone previous surgery, 49 had a central venous catheter, and only 13 had been treated with immunosuppressants. Predominant underlying pathologies were chronic renal failure, chronic obstructive pulmonary disease, solid tumor, and diabetes mellitus (24, 22, 21, and 17 patients, respectively). Thirty-nine experienced septic shock. Most patients received anidulafungin or caspofungin for antifungal treatment. *C. albicans* was the prevalent isolated species (51.1%), followed by *C. parapsilosis* (23.9%), *C. tropicalis* (9.8%), *C. glabrata* (7.6%), and several other minor species (in total 7.6%).

None of the patients had bacterial co-infection at the time of candidemia diagnosis.

Thirty-eight of these patients (41.3%) did not survive within 30 days from the first documented episode of candidemia.

### Antibodies against the Mp65, Als3, Hyr1, and Eno1 proteins

**Fig 1:**
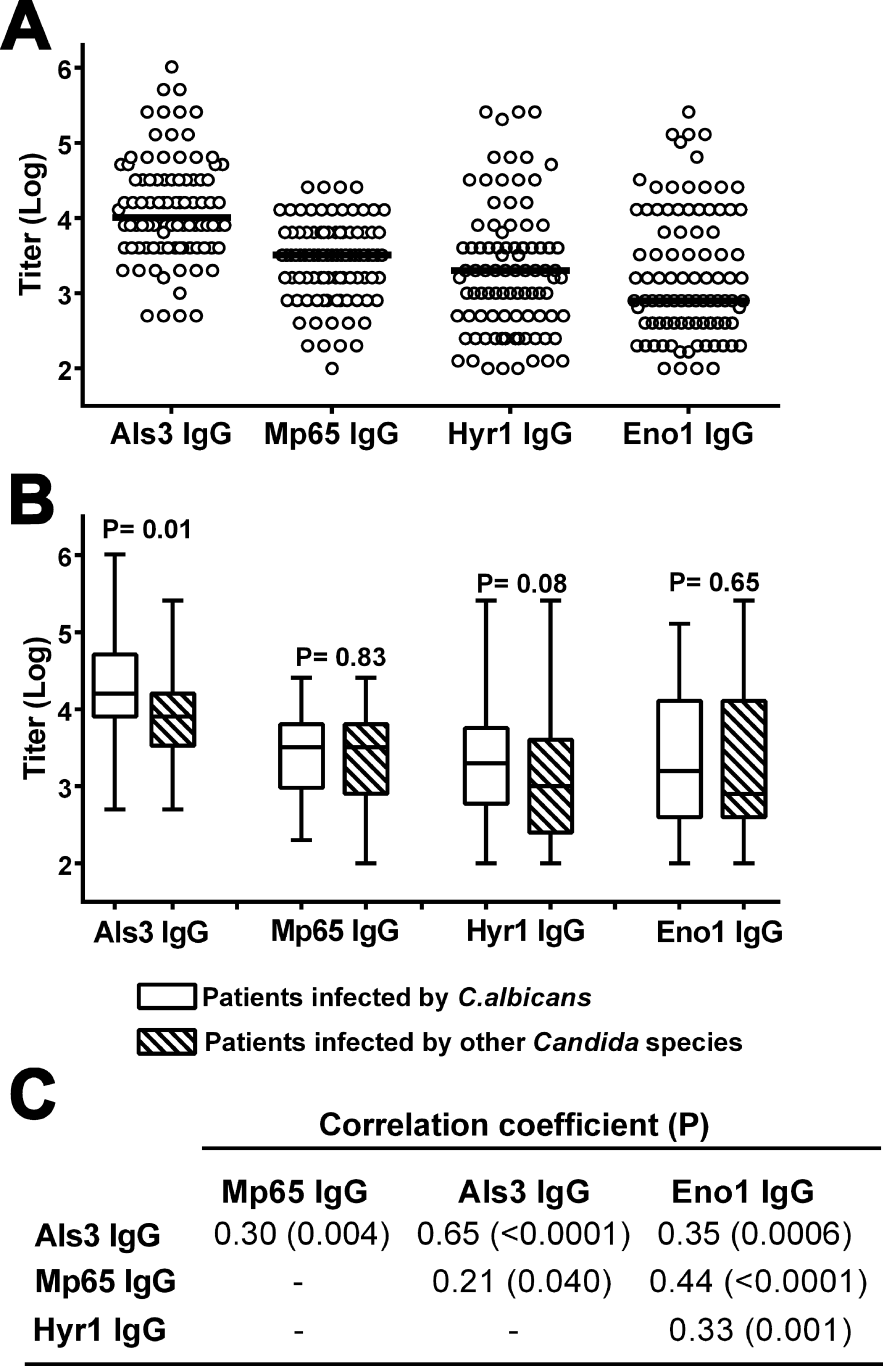
Levels of Als3- Mp65-, Hyr1- and Eno1-reactive IgG in candidemia patients. (A) Individual IgG titers against the different *Candida* proteins in candidemia patients (*n* = 92). Titer values in the graph are log_10_-trasforms of reciprocal serum titers as evaluated in ELISA (see Materials and Methods). The thick lines indicate median values. (B) Box and whisker plot comparing titer ranges toward the Als3, Mp65, Hyr1, and Eno1 antigens in patient infected by *C. albicans* (*n* = 47) or by other *Candida* species (*n* = 45). Probability (*P*) was calculated by the Mann-Whitney *U* test. (C) Correlations among patient IgG responses toward the investigated *Candida* proteins. Correlation coefficient and probability (*P*) were evaluated by the Spearman’s rank correlation test.

The antibody responses against the selected *Candida* proteins were evaluated in sera of all patients with candidemia by indirect ELISA, using recombinant Mp65, Als3, Hyr1 or Eno1 as the solid phase antigen. A single serum sample, derived from the blood specimen used for the first *Candida*-positive blood culture, was examined for each patient. As shown in Fig. 1, substantial levels of IgG specific to each tested protein were found. Antibody titers (log_10_) ranged from 2.7 to 6.0 (median value 4.0), 2.0 to 4.4 (median 3.5), 2.0 to 5.4 (median 3.3), and 2.0 to 5.4 (median 2.9), for the anti-Als3, Mp65, Hyr1, and Eno1 IgG, respectively (Fig.1A). The levels of IgM toward all protein antigens were negligible in all patients (not shown). IgG titers (ranges) against the Eno1 and Mp65 antigens, which are expressed by both the yeast and filamentous forms of *C. albicans*, were not significantly different in patients with candidemia due to *C. albicans* and those infected by other *Candida* species (Fig. 1B). In contrast, titers against the Als3 and Hyr1 antigens, which are selectively expressed by the filamentous forms of *C. albicans*, were higher in patients with candidemia due to this species. The IgG against Als3 and Hyr1 or those against Mp65 and Eno1 were also the antibodies that most significantly correlated with each other (Fig. 1C ). As expected from a previous investigation restricted to Mp65 (8), the ranges of antibody titers against all the four antigens were significantly higher in patients, irrespective of the infecting *Candida* species, than in control subjects with risk factors of candidemia but without candidemia diagnosis, confirming the data of a previous investigation (Fig. S1; see also reference 8) for details about the above risk factors].

### Antibody immunity and survival of candidemia patients

We next investigated the possible relationship between antibody levels and candidemia outcome, defined as patient survival or death within 30 days from candidemia diagnosis. In a first analysis, we classified the candidemia patients into three groups defined as “low” (patients with IgG titer values up to the 25th percentile), “medium” (titers between the 25th and 75th percentile), or “high” (titers higher than the 75th percentile) and assessed the percentage relative frequency of survivors and non-survivors in the three classes.

**Fig 2:**
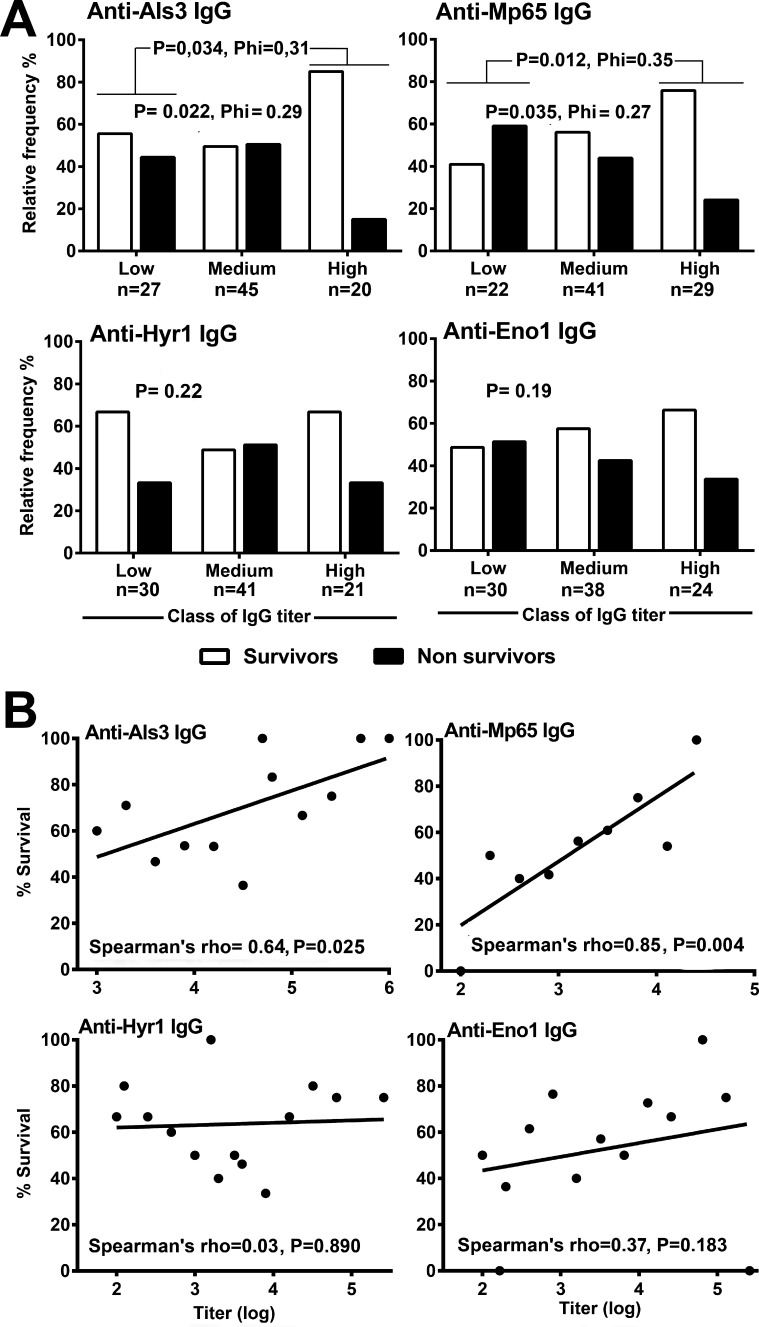
Relationships between magnitude of the anti-Als3, Mp65, Hyr1, or Eno1 IgG responses at onset of candidemia and patient survival. (A) Relative frequency of survivors and non-survivors in the classes of candidemia patients showing low, medium, or high serum titers of anti-Als3, Mp65, Hyr1 ,or Eno1 IgG. Phi and probability (*P*) values are from chi-squared tests comparing number of survivors and non-survivors in the three different titer classes or between patients of the low and the high class. Patient titer classes were defined as reported in Materials and Methods. (B) Correlations between anti-Als3, Mp65, Hyr1, or Eno1 antibody titers and patient survival rates. The graphs show linear regression analyses of percentage of survived patients at all the different antibody titer levels. Rho = Spearman’s rank correlation coefficient; *P* = probability.

The data (Fig. 2A) showed a significantly increased frequency of survivors from the “low” to the “high” antibody titer class for the anti-Als3 and Mp65 IgG (*P* = 0.035 and *P* = 0.022, respectively, from a chi-squared test comparing the number of survivors and non-survivors in the three different classes, and *P* = 0.034 and 0.012, respectively, comparing directly the “low” with the “high” antibody class). In contrast, the frequency of survivors and non-survivors in the different titer classes did not significantly differ for the anti-Hyr1 and anti-Eno1 IgG (*P* > 0.1 by all chi-squared tests). We then asked whether antibody titers correlated with the percentage of survival, using Spearman’s rank correlation tests. As shown by the scatter plots in Fig. 2B, the two variables were significantly correlated only in the case of anti-Mp65 (*P* = 0.004; rho = 0.85; 95% CI 0.43–0.97) and anti-Als3 antibodies (*P* = 0.025; rho = 0.64; 95% CI 0.11–0.88)

To evaluate the association between anti-Als3 and/or anti-Mp65 antibodies and candidemia outcome, we performed multivariable logistic regression analyses adjusted for potential confounding factors (Table 2). The data confirmed that Als3 and Mp65 antibody levels are independent predictors of survival (Als3 IgG: adjusted odds ratio 3.10, 95% CI 1.3–7.4; Mp65 IgG: adjusted OR 3.47, 95% CI 1.2–10.2). Other significant, outcome (negative) predictive variables were the highest serum β-glucan levels at diagnosis of candidemia, the occurrence of septic shock, the presence of diabetes, the initial antifungal treatment with fluconazole (compared with all other used antimycotics) and, weakly, infection by the *C. albicans* species, and an older age.

**TABLE 2:** Factors correlated to survival/non survival of patients with candidemia^*a*^

		Anti-Als3 IgG			Anti-Mp65 IgG	
**Independent variable**	Coeff	*P*	OR (95% Cl)	Coeff	*P*	OR (95% Cl)
Antibody titer	1.13	0.011*	3.10 (1.3–7.4)	1.25	0.021*	3.47 (1.2–10.2)
Diabetes	−1.78	0.014*	0.17 (0.04–0.7)	−1.61	0.024*	0.20 (0.05–0.8)
Level of β-glucanemia	−1.10	0.005*	0.33 (0.15–0.72)	−0.95	0.013*	0.39 (0.18–0.82)
Septic shock	−2.16	0.0004*	0.11 (003–0.37)	−1.82	0.001*	0.16 (0.05–0.48)
Initial therapy with fluconazole	−1.58	0.037*	0.20 (0.05–0.91)	−1.51	0.048*	0.22 (0.05–0.98)
Infection by *C. albicans*	−1.02	0.086	0.36 (0.11–1.5)		ni	
Age		ni		-0.03	0.10	0.97 (0.93-1.01)
	Overall model fit, *P*<0.0001 AUC= 0.86 (0.77-0.92 95% Cl)			Overall model fit, *P*<0.0001 AUC= 0.85 (0.76-0.91 95% Cl)		

^*a*^ Data are from multivariate logistic analyses for prediction of 30-day survival, each taking into account as independent variables all demographic, laboratory, and clinical variables of patients (Table 1) and serum titers of a single anti-*Candida* antibody, as indicated. Final logistic models, constructed using the “backward” algorithm for variable selection (cutoff for inclusion in the final model: *P* < 0.1), are shown in the table. Anti-Hyr1 and anti-Eno1 IgG titers are not associated to survival and are not mentioned. Number of subjects = 92. Coeff, coefficient; OR, odds ratio; 95% Cl, 95% confidence limits; AUC, area under the curve; ni, variable not included in the final model (*P* > 0.1). * *The asterisks indicate *P* < 0.05.

### Anti-*Candida* antibodies and survival in different groups of candidemia patients

We then collectively evaluated by multivariable logistic regression analyses the survival predictive values of anti-Als3, Mp65, Hyr1, and Eno1 IgG levels in the entire cohort of patients or in patient subgroups with or without risk factors for mortality. To this aim, logistic models were constructed which accounted for the antibody titers against all the four antigens. As shown in Table 3, the anti-Als3 IgG was the only significant survival-predictive antibody variable in the entire study cohort and in all subgroups of more critical patients, such as those of over-median age (>72), infected by the most pathogenic species *C. albicans*, or with septic shock. On the other hand, the anti-Mp65 IgG levels resulted the only antibody variable significantly associated to patient survival in all counterpart groups (patients aged ≤72, infected by non-albicans *Candida* species or showing no signs of septic shock). Here again, the anti-Hyr1 and anti-Eno1 IgG titers showed no relationship with the candidemia outcome, with values of *P* > 0.1 in all patient groups (not shown).

**TABLE 3:** Relationships between antibody levels to the Als3 and Mp65 antigens and 30-day survival in different subgroups of patients^*a*^

Group	Variables			
	Anti-Als3 titer	Anti-Mp65 titer	Other variables in the model (OR, ***P***)	
All patients (*n* = 92)	OR = 3.10 (1.3–7.4 95% CI) *P*= 0.011*	OR = 2.46 (0.7–8.2 95% CI) *P* = 0.10	Diabetes (0.17, 0.014*) Glucanemia (0.33, 0.005*) Septic shock (0.11, 0.0004*) Fluconazole (0.21, 0.037*	AUC = 0.858 (0.77–0.92 95% Cl) *P* (*F* test) < 0.0001*
Septic shock (*n* = 32)	OR = 4.34 (1.3–14.0 95% CI) *P*= 0.019*	ni	No other variable	AUC = 0.746 (0.58–0.87 95% Cl) *P* (*F* test) = 0.009*
Age > 72 (n=46)	OR = 10.0 (1.6–42 95% CI) *P*= 0.013*	ni	*C. albicans* (0.22, 0.085) APACHE (0.88, 0.029*) Glucanemia (0.33, 0.046*) Caspofungin (3,3, 0.036*)	AUC = 0.839 (0.70–0.93 95% Cl) *P* (*F* test) = 0.001*
*C. albicans (n*=47)	OR = 5.11 (1.3–21 95% CI) *P*= 0.024*	ni	Diabetes (0.12, 0.034*) Glucanemia (0.34, 0.045*) Septic shock (0.08, 0.012*) Fluconazole (0.094,0.058)	AUC = 0.815, (0.73–0.94 95% Cl) *P* (*F* test) = 0.0007*
No septic shock (*n* = 53)	ni	OR = 4.41 (1.2–18 95% CI) *P*= 0.037*	Glucanemia (0.13, 0.036*) Chronic renal failure (0.26, 0087)	AUC = 0.841 (0.71–0.93 95% Cl) *P* (*F* test) = 0.0016*
Age ≤ 72 (*n* = 46)	ni	OR = 13.4 (1.9–57 95% CI) *P*= 0.001*	Septic shock (0.08, 0.013*) Antibiotics (3.3, 0.059)	AUC = 0.885 (0.75–0.93 95% Cl) *P* (*F* test) = 0.0002*
Non-*albicans* species (*n* = 45)	ni	OR = 6.42 (1.2–34 95% Cl) *P*= 0.031*	Diabetes (0.12, 0.085) Glucanemia (0.09, 0.025*) Fluconazole (0.096, 0.070)	AUC = 0.849 (0.71–0.91 95% Cl) *P* (*F* test) = 0.003*

^*a*^ The table reports complete logistic regression models for prediction of survival in the indicated patient groups. Independent variables initially considered were those reported in Table 1 and titer values of all four investigated anti-*Candida* antibodies. Model variables were selected using the “backward” algorithm, with cut-off for model inclusion at *P* < 0.1. OR, odds ratio; 95% Cl, 95% confidence limits; AUC, area under curve; ni, not included in the final model (*P* > 0.1). Anti-Hyr1 and anti-Eno1 IgG showed no association to survival (*P* > 0.1) in all patient groups and are not mentioned in the table. * The asterisks indicate *P* < 0.05.

While generally confirming the positive relationship between the increase of anti-Als3 or anti-Mp65 IgG titers and survival, our data also suggest that the two antibodies are not equivalent survival predictors but could be differentially involved in different patient conditions and/or severity of the infection.

### Patient factors that modulate the anti-Als3 and anti-Mp65 antibody response

We finally investigated whether there were patient conditions influencing the antibody response to Als3 or Mp65, particularly in those patients with risk factors of a more severe infection and/or higher mortality (over-median aged >72, with over-median APACHE II score > 15, or undergoing septic shock).

Multivariable linear regression analysis showed that higher APACHE II score, infection by *C. albicans*, treatment with steroids, and, mostly, treatment with immunosuppressants (particularly when associated with steroids) significantly correlated with increasing levels of anti-Als3 antibodies (Table 4).

**TABLE 4:** Patient factors affecting the magnitude of the IgG response to the Als3 or Mp65 antigen at the onset of candidemia^*a*^

	Dependent variable: titer of anti-Als3 IgG							
	**All patients** **(** ***n*** **=92)**		**Patients with APACHE score >15 (** ***n*** **=41)**		**Patients aged >72** **(** ***n*** **=46)**		**Septic shock patients** **(n=39)**	
**Independent variable**	*r* partial	*P*	*r* partial	*P*	*r* partial	*P*	*r* partial	*P*
*C. albicans*	0.27	0.009*	0.27	0.10	0.46	0.002*	0.48	0.0036*
APACHE score	0.22	0.038*	0.39	0.001*	0.35	0.023*	0.52	0.0013*
Immunosuppressants + steroids	0.29	0.005*	0.47	0.003*	0.45	0.002*	0.59	0.0002*
Immunosuppressant	ni		0.35	0.035*	0.41	0.007*	0.45	0.006*
Diabetes	−0.18	0.096	ni		−0.27	0.081	ni	
Steroids	ni		0.32	0.049*	ni		ni	
	*R*^2^ = 0.21, *R*^2^Adj = 0.17 *P* (*F* test) = 0.0003		*R*^2^ = 0.45, *R*^2^Adj = 0.37 *P* (*F* test) = 0.0006		*R*^2^ = 0.45, *R*^2^Adj = 0.37 *P* (*F* test) = 0.0002		*R*^2^ = 0.59, *R*^2^Adj = 0.53 *P* (*F* test) < 0.0001	

	Dependent variable: titer of anti-Mp65 IgG							
	**All patients** **(** ***n*** **=92)**		**Patients with APACHE score** **>15 (** ***n*** **=41)**		**Patients aged >72** **(** ***n*** **=46)**		**Septic shock patients** **(** ***n*** **=39)**	
**Independent variable**	*r* partial	*P*	*r* partial	*P*	*r* partial	*P*	*r* partial	*P*
Diabetes	−0.28	0.008*	ni		ni		ni	
Hematologic malignancy	−0.19	0.069	−0.31	0.055	ni		ni	
Level of glucanemia	ni		ni		0.41	0.005*	ni	
Previous surgery	ni		ni		−0.33	0.026*	ni	
Immunosuppressants+ steroids	ni		0.28	0.075	ni		ni	
Age	ni		ni		ni		0.27	0.092
	*R*^2^ = 0.11, *R*^2^Adj = 0.086 *P* (*F* test) = 0.007		*R*^2^ = 0.24, *R*^2^Adj = 0.12 *P* (*F* test) = 0.008		*R*^2^ = 0.16, *R*^2^Adj = 0.19 *P* (*F* test) = 0.033		*R*^2^ = 0.07, *R*^2^Adj = 0.05 *P* (*F* test) = 0.092	

^*a*^ The table shows the final, multiple linear regression models for the different, indicated patient groups. The analyses initially considered as independent variables a number of patient factors likely to impact the antifungal immune response (age, APACHE II score, beta-glucanemia at diagnosis, infection by *C. albicans*, presence of diabetes, solid tumor or hematologic malignancy, previous surgery and treatment with steroids, immunosuppressive drugs, or both), and titer values of the indicated antibody as the dependent variable. Selection of independent variables to be included in the model was performed by the “backward” algorithm (cutoff for inclusion: *P* < 0.1). Part *r*, partial coefficient of determination; *R*^2^, coefficient of multiple determination of the model; *R*^2^Adj, adjusted coefficient of multiple determination; ni, variable not included in the model (*P* > 0.1). * Significant *P* values (<0.05) are indicated by asterisks.

The positive impact of these factors on the magnitude of the anti-Als3 antibody response was limited when considering the total patient cohort (adjusted multiple determination coefficient [*R*^2^Adj] of the model = 0.17) but was quite more pronounced when referred to frail patients, such as those with over-median APACHE II score (*R*^2^Adj = 0.37), over-median age (*R*^2^Adj = 0.37), or septic shock (*R*^2^Adj = 0.53). In particular, the variable “treatment with steroids + immunosuppressants” yielded a partial coefficient of determination (part *r*) of 0.29 in the entire cohort that increased to 0.47 and 0.45 in patients with APACHE II score > 15 or aged >72, respectively, and rose up to 0.59 in septic shock patients. In contrast, anti-MP65 IgG levels were only weakly and negatively correlated with the presence of diabetes, hematologic malignancies, or previous surgery and directly correlated with β-glucan levels in patients aged >72.

**Fig 3:**
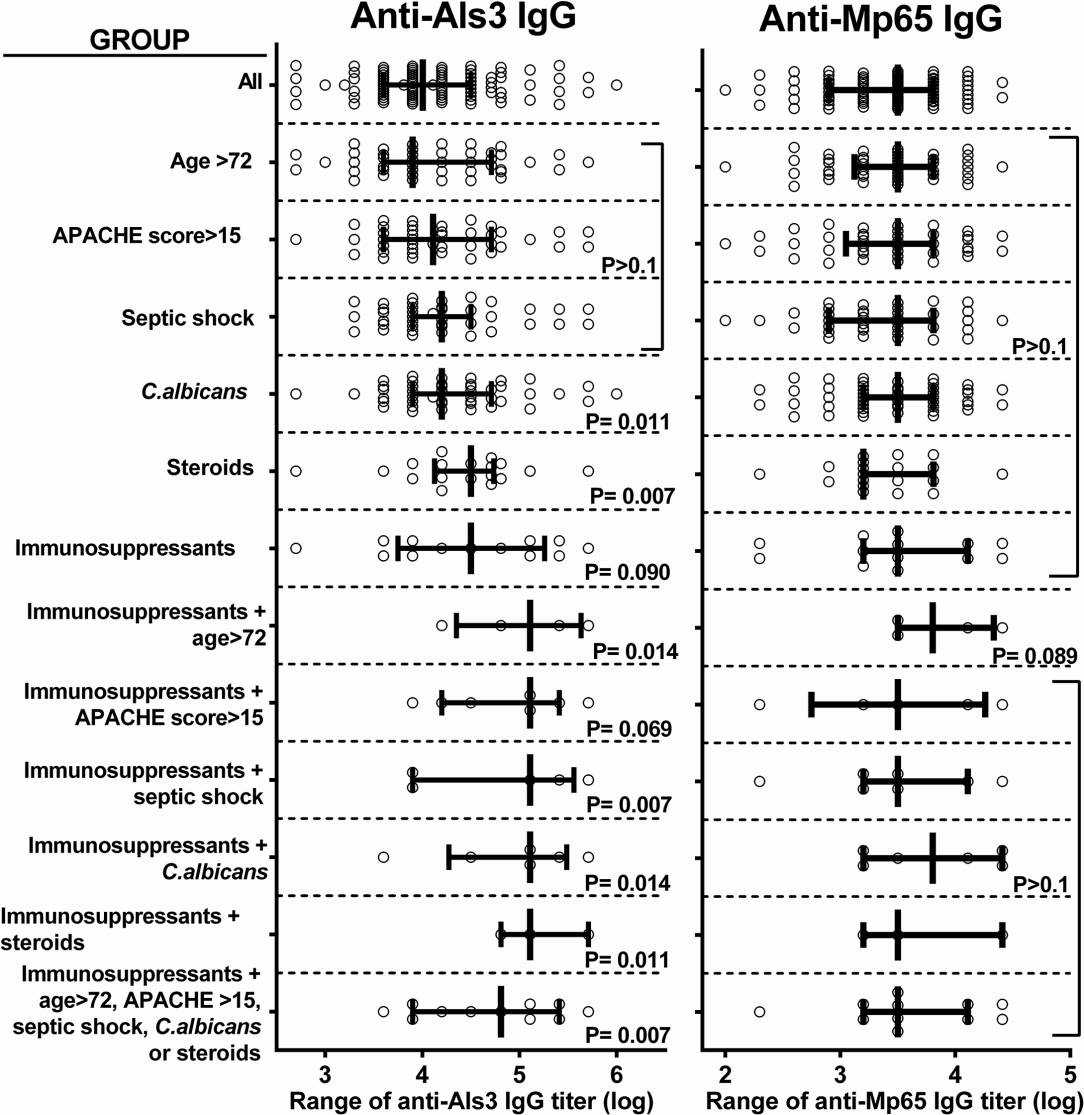
Combination of immunosuppressant treatment with other risk factors for severe disease enhances anti-Als3, but not anti-Mp65, antibody response at candidemia onset. The figure shows antibody titer distributions in the indicated groups of patients. The central lines mark median values, left and right lines the 25th and 75th percentile, respectively. *P* values were calculated by Mann-Whitney *U* tests comparing IgG titer range of the relative patient group with that of all other patients of the cohort.

These results were confirmed by direct analysis of anti-Als3 or Mp65 levels in subgroups of frail, immunosuppressant-treated patients (Fig. 3).

Thus, based on these analyses, Als3-reactive, but not Mp65-reactive, IgG appear to be more elevated in patients with certain risk factors for severe candidemia or mortality.

## DISCUSSION

Candidemia continues to be a life-threatening disease despite improvements in diagnosis and the availability of some effective therapeutics (1, 2, 4). No vaccines or other immunological tools are presently available for candidemia prevention or treatment. Nonetheless, the general non-immunocompromised patient status and clear evidence from experimental animal models of invasive candidiasis suggest that both cellular and humoral host responses could play a role for candidemia control (5). Vaccines based on recombinant cell wall antigens of *Candida albicans* have repeatedly been shown to confer protection against disseminated or local infection by the above fungus in mice through the induction of specific, protective antibodies (recently reviewed in reference 10).

In this context, we have previously reported that antibody responses of candidemia patients to the Mp65 protein are boosted by the infecting *Candida* species, and high anti-Mp65 IgG titers were found in the patients that survived candidemia, suggesting the activation during candidemia of a potentially protective memory response early primed by *Candida* commensalism (8).

In the present paper, we expanded our investigation to other well-known *Candida* antigens to assess whether the apparently protective response to Mp65 was a peculiar, unique phenomenon or antibodies to other antigens could also be involved. The additional antigens included two proteins (Als3 and Hyr1) that have been shown to be associated with the filamentous, tissue-invasive forms of *Candida albicans*, a major candidemia agent, and the Eno1 protein that, like Mp65, is expressed by both the yeast and the filamentous forms (11, 12). The relationship between the above antibodies and the disease outcome was analyzed using correlation and multivariable logistic regression analyses to determine the impact of these antibodies on survival in the entire cohort and in groups of patients with distinct candidemia risk factors.

The main result of this study is the finding of a statistically significant and sizeable association of antibodies to Als3 and Mp65 with patient survival. In particular, multivariable logistic regression analyses demonstrated that these antibodies are independent, positive outcome-predictive variables, while patient variables such as diabetes, high β-glucanemia levels, and septic shock are predictive of a negative outcome.

As expected from previous literature data and the IgG antibody class (8, 13, 14), we here confirm that the candidemia patients had apparently mounted a memory antibody immunity toward all tested proteins upon *Candida* infection. In fact, their IgG levels to each protein were significantly higher, irrespective of the infecting species (*C*. *albicans* or non-*albicans*) than in non-candidemia control subjects. This conclusion is also indirectly corroborated by the observation that in patients infected by the filamentation-competent species *C. albicans*, the IgG titers against the hypha-associated antigens Als3 and Hyr1 were higher than in patients infected by other *Candida* species, in which these antigens are less represented (11). Together with the absence of an IgM response at the candidemia diagnosis, our data confirm and expand a previous report (8), strongly suggesting that the IgG elevation in patients with candidemia is a genuine memory response.

The capacity of mounting a sustained secondary antibody response despite the presence of several known risk factors for lethal candidemia such as advanced age, diabetes, high APACHE score, and the use of corticosteroids and immunosuppressants, which are generally expected to dampen rather than stimulate immune responses, is noticeable but not surprising. Memory B cells can indeed recognize the antigen and produce large antibody quantities upon restimulation by *Candida* infection. Rather unexpected, however, is the finding that some of the above risk factors could be associated to the enhancement of the antibody response to only specific fungal antigens, as observed for the anti-Als3 antibodies (see also below). Although the reasons for elevated titers of Als3-reactive antibodies in frail patients are unknown, clinical frailty does not necessarily imply an absence of immune competence, particularly as a response to a presumably large burden of invasive, Als3-expressing fungal cells. Nonetheless, more work is needed to explain this finding.

We also noticed that the association between anti-Mp65 or Als3 antibodies and survival is antigen dependent in different categories of candidemia subjects. In particular, increased anti-Als3 IgG levels were the predominant survival-predictive antibody factor in severely ill, high-mortality risk patients, including those with septic shock, with over-median age, or infected by the more pathogenic species *C. albicans*, whereas increased levels of anti-Mp65 IgG were predictive of a positive outcome in patients with milder infection/lower mortality risk. It is unlikely that hyphal development is the only determinant of this phenomenon, as elevated antibodies against Hyr1, which is also a typical hyphal antigen and has here shown antibody levels highly correlated with the anti-Als3 ones, are not associated with a better candidemia outcome. It has been shown that mouse sera enriched with anti-Hyr1 antibodies potentiate fluconazole therapy in a model of disseminated candidiasis of neonate mice (15). However, a mouse model cannot be compared with human candidemia. The different magnitudes of the antibody responses against Als3 and Hyr1 in our candidemia patients should also be considered to possibly play a role. In fact, the median anti-Als3 antibody titers were about one log higher than those against the Hyr1 antigen, both in the entire study cohort and in all patient subgroups.

It therefore appears that both memory antibody responses and the association of the latter with candidemia survival could be variable depending on the targeted antigen, the response magnitude, and the underlying conditions of the patients with their risk profiles, a finding that is coherent with the multifactorial nature of this opportunistic disease.

The reasons for the apparent protective role of some high titer anti-*Candida* antibodies are not known. Both Als3 and Mp65 have been shown to function as *Candida* virulence adhesins in valuable experimental models of *Candida* infection (16–27). A Als3 preparation gave favorable results in a human trial of vaccination of women with chronic, recurrent candida vaginitis (28), with the anti-Als3 antibodies being reported as surrogate markers of protection (29). At least for candidemia caused by *C. albicans*, Als3 antibodies could somehow affect hyphal growth and consequently reduce the fungus invasive capacity. In addition, immunocomplexes with Mp65 and/or Als3 antigens could activate the classical complement pathway, leading to the generation of complement fractions capable of promoting fungal phagocytosis or extracellular killing by the phagocytes. In this line, noteworthy is the recent report of a suggested role of the C5a complement fraction in promoting the survival of patients with candidemia (30). Of note, anti-Hyr1 and anti-Eno1 antibodies were not associated with enhanced survival, although both of these proteins have also been considered to be somehow related to *Candida* virulence (31–34). It is also possible that anti-Als3 and anti-Mp65 antibodies are surrogate markers of other, unknown protective factors related to these proteins, such as, for instance, Als3- and Mp65-directed T cell-mediated responses, as suggested by Spellberg et al. in a murine model of experimental invasive candidemia upon vaccination with the recombinant N-terminus of the Als3 protein (35). Further investigations are needed to clarify this issue.

Our study has several limitations. It is retrospective and monocentric in nature; consequently, its results could be affected by several biases which could not be eliminated or reduced. These limitations are somewhat tempered by the selectivity of the apparently protective antibody response and the examination of a relatively high number of candidemia subjects at a large clinical center. It is also comfortable that the logistic models employed, while identifying anti-Als3 and anti-Mp65 antibodies as associated with a better prognosis, confirmed some of the well-known demographic, clinical, and therapeutic factors that are associated with a negative candidemia prognosis. Nonetheless, the magnitude of the impact of antibody titers on patient survival has a degree of uncertainty because of the generally wide confidence intervals of the effect size estimators used in the study, possibly due to the limited number of patients.

In addition, the quantity of serum available for investigation was very low and did not allow for repeated testing that could help data interpretation and mechanistic studies. For all the above reasons and as previously stated (8), the conclusions of our study should be taken cautiously and mostly considered as a strong indication for further prospective and possibly multicentric new studies whereby firmer evidence for the protective role of some anti-*Candida* antibodies in patients with candidemia could be gained. In conclusion, our study reports novel findings that comprehensively demonstrate that some anti-*Candida* antibodies can have an impact on candidemia outcome and could be considered independent prognostic factors in patients affected by this severe fungal disease.

## MATERIALS AND METHODS

### Study population and design

We included in the study patients with culture-proven candidemia who were hospitalized from January 2012 to February 2016 at the A. Gemelli Hospital of Rome, Italy, an academic tertiary care center with 1,400 beds and ~50,000 hospital admissions per year. Patients were identified by electronically querying the clinical microbiology laboratory database and were included only if complete data series (as reported in Table 1) could be retrieved from their medical charts and from laboratory databases. All patient samples and data records were rendered anonymous before performing any experimental or statistical analyses.

The study was approved by the local institutional review committee, and informed consent was waived because of the observational, retrospective nature of the investigation.

Diagnosis of candidemia was made on the basis of at least one blood cultures growing *Candida* species, positive β1,3-glucanemia assay (>80 pg/mL), and on the presence of signs and symptoms of infection. Only the first episode of candidemia was reported for patients with recurrent or subsequent episodes of infection.

Non-candidemia controls were age- and sex-matched patients who were admitted to the hospital within the same time interval as the candidemia ones and considered at risk of candidemia but found with *Candida*-negative blood culture and negative β-glucanemia (see reference 8 for other details about the controls)

### Microbiological assays

Blood cultures were obtained as part of normal clinical practice and processed using a Bactec (BD Diagnostic Systems, Sparks, MD) or BacT/Alert (bioMérieux, Marcy l’Etoile, France) system. After subculturing on Difco *Candida* bromocresol green (BCG) agar medium, yeast isolates were identified to the species level by matrix-assisted laser desorption ionization-time of flight mass spectrometry (36). Sera from blood specimens of patients were tested for the presence of β1,3-D-glucan by the Fungitell assay (Associates of Cape Cod Inc., Falmouth, MA; range of the assay from 31.25 to 500 pg/mL), as previously described (37). The assay was considered positive if the value was ≥80 pg/mL, according to the cutoffs proposed by the manufacturer.

### Evaluation of the antibody titers against the Als3, Mp65, Hyr1, and Eno1 protein in patients’ sera

A single serum sample was examined from each subject under study which, for candidemia patients, corresponded to laboratory diagnosis of the first candidemia episode (i.e., obtained from the same blood specimen of the first *Candida*-positive blood culture). All sera were heated at 56°C for 30 min and stored individually at −80°C before the analysis.

Full-length, poly-histidine tagged, recombinant protein portion of cell surface mannoprotein MP65 (Mp65) and enolase1 (Eno1) were produced in *Escherichia coli* by GenScript (Piscataway, NJ) and purified to homogeneity by nickel-affinity HPLC. GMP-grade, full-length, recombinant agglutinin-like protein 3 (Als3) and hyphally regulated protein 1 (Hyr1) were a gentle gift from Dr PL Hennessey and JE Edwards, NovaDigm, Colorado, US.

Immunoglobulin G (IgG) and M (IgM) titers against the protein antigens under study were determined by ELISA, essentially as already described (8). Briefly, polystyrene microtiter plates (MaxiSorp; NUNC, Roskilde, Denmark) were coated with the antigen (0.5 µg/mL protein in 0.05 M carbonate buffer, pH 9.6, 100 µL/well, overnight at +4°C) and blocked 2 h at 37°C with Blocker Casein in PBS (Thermo Scientific, Rockford, IL, USA). Wells were then reacted with twofold dilutions of the human sera in blocking solution, followed by affinity-purified, gamma chain-specific, goat anti-human IgG or mu chain-specific, goat anti-human IgM conjugated to alkaline phosphatase (Merck-Sigma Aldrich, Darmstadt, Germany). Plates were developed 30 min with the enzyme substrate p-nitrophenyl phosphate disodium (Merck) and read for absorbance at 405 nm. Readings from negative control wells (wells without antigen) were subtracted from all absorbance values. Antibody titers were defined as the reciprocal of the highest dilution of sera that gave an optical density at least twice that of the correspondent negative control.

### Statistics

All statistical analyses were performed with the MedCalc software v20.022 (MedCalc Software Ltd., Ostend, Belgium).

Antibody titers of patients were log_10_ transformed before all the analyses. Comparison of antibody titer among groups was performed with the Mann-Whitney *U* test. Spearman’s rank correlation test was used to assess the relationship among antibody titers to the different fungal antigens as well as the correlations between magnitude of antibody titers and percentage of survived patients.

Multivariate logistic regression models for survival were constructed initially considering all available independent variables of patients and their antibody titers (against all tested proteins or against a given protein only, as specified in “Results” and table legends). Variables to be included in the final models were selected with the use of the backward algorithm, setting the threshold significance of a variable to enter the model at *P* < 0.1.

Multiple linear regression analyses with the use of the backward algorithm were performed to analyze the influence of some potentially immune response-modifying patient conditions on the antibody response against the investigated proteins. In these analyses, variables again entered the final models if their *P* was <0.1, and VIF values were always <1.5.

The effect size was estimated by calculation of Cramer’s phi, Spearman’s rank correlation coefficient rho, AUC, or adjusted *R*^2^, depending on the relevant analysis (chi-squared test, correlation test, multivariable logistic or linear regression).

In some analyses, the patient cohort was subgrouped according to presence/absence of a condition, or according to the variable value (up to the median value of the entire cohort/higher than the median) for continuous variables such as age or APACHE score.

Only for chi-squared test analyses, antibody titers against each antigen were categorized into three classes based on their value distribution (low, approximately up to the 25th percentile; medium, between the 25th and 75th percentile; and high, higher than the 75th percentile). The three categories included 29.3% (low), 48.8% (medium), and the 21,8% (high) of patients for the anti-Als3 antibodies; 23.9%, 44.6%, and 31.5% of patients for the anti-Mp65 antibodies; 32.6%, 44.6%, and 22.8% of patients for the anti-Hyr1 antibodies; and 32.6%, 41.3%, and 26.1% of patients for the anti-Eno1 antibodies.

In all the above analyses, a *P* < 0.05 was accepted as statistically significant.

## SUPPLEMENTAL MATERIAL

Figure S1 mbio.02769-23-s0001.tif Comparison of serum IgG titers to Als3, Mp65, Hyr1 and Eno1 in patients with or without candidemia.

Legend mbio.02769-23-s0002.docx Legend to Fig. S1.

ASM does not own the copyrights to Supplemental Material that may be linked to, or accessed through, an article. The authors have granted ASM a non-exclusive, world-wide license to publish the Supplemental Material files. Please contact the corresponding author directly for reuse.
