# Supplementary figures and images for: Identification of two anti-Candida antibodies associated with the survival of patients with candidemia
Source: mBio. 2023 Dec 13;15(1):e02769-23. doi: 10.1128/mbio.02769-23 (PMC10790786; doi:10.1128/mbio.02769-23)

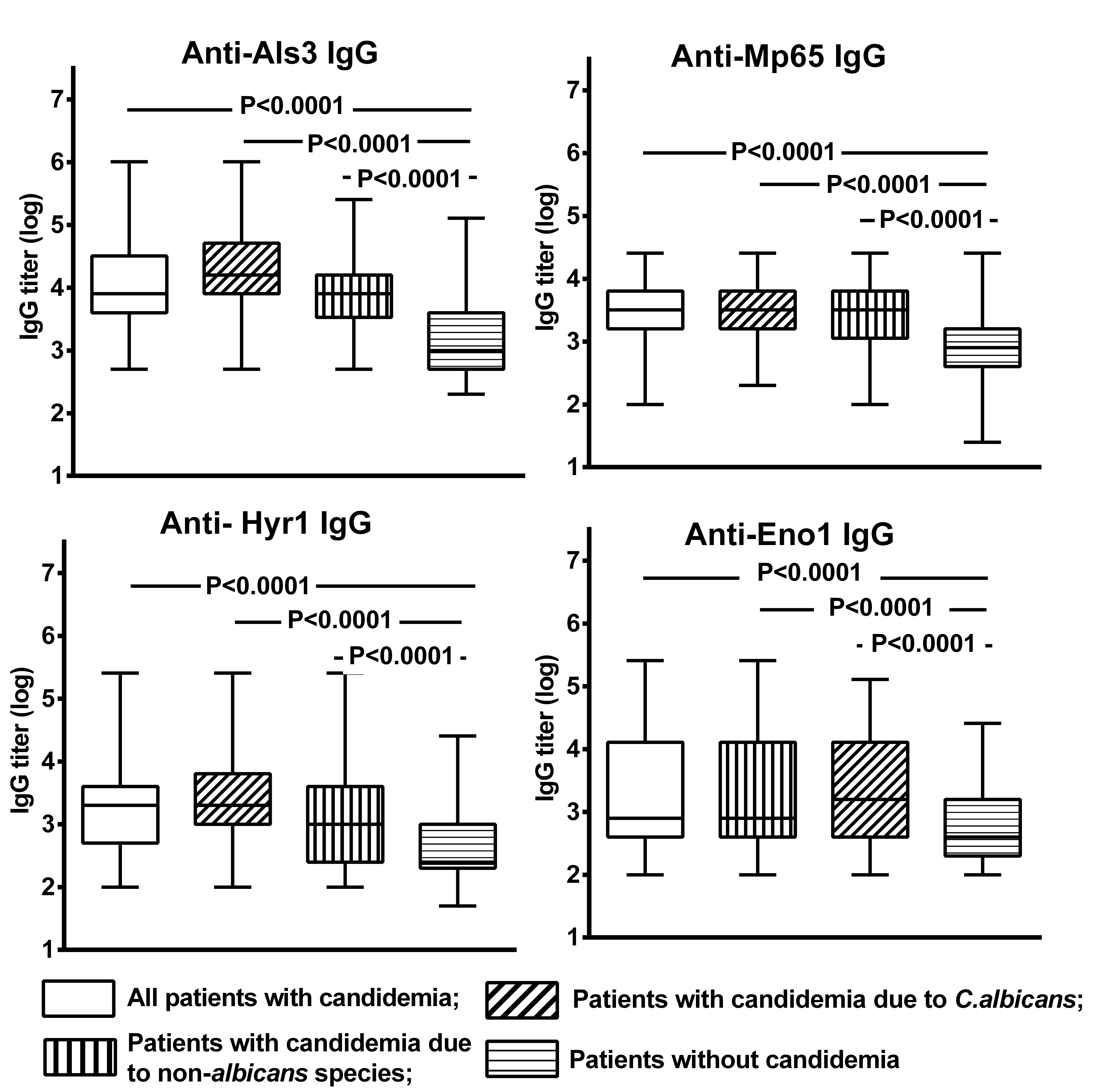

Supplement: Figure S1 — Comparison of serum IgG titers to Als3, Mp65, Hyr1 and Eno1 in patients with or without candidemia. [file mbio.02769-23-s0001.tif]
