# Supplementary material for: Identification of two anti-Candida antibodies associated with the survival of patients with candidemia
Source: mBio. 2023 Dec 13;15(1):e02769-23. doi: 10.1128/mbio.02769-23 (PMC10790786; doi:10.1128/mbio.02769-23)
Supplement: Legend — to Fig. S1. [file mbio.02769-23-s0002.docx]

Figure S1. Comparison of serum IgG titers to Als3, Mp65, Hyr1 and Eno1 in patient with or without candidemia.

The graph compares the ranges of antibody titers measured in the whole cohort of candidemic patients, in patients infected by *C.albicans* or in those infected by non-*albicans* species, with the titer ranges measured in a group of patients (n=134) hospitalized in the same period and in the same wards as the candidemic ones, who were considered at risk for candidemia but found not infected (see Methods and Torosantucci A, Tumbarello M, Bromuro C, Chiani P, Posteraro B, Sanguinetti M, Cauda R, Cassone A. 2017. Sci Rep 7:2722)
